# Carotid Arterial Stiffness and Cardiometabolic Profiles in Women with Fibromyalgia

**DOI:** 10.3390/biomedicines9121786

**Published:** 2021-11-28

**Authors:** Yunkyung Kim, Geun-Tae Kim, Jihun Kang

**Affiliations:** 1 Division of Rheumatology, Department of Internal Medicine, Kosin University Gospel Hospital, Kosin University College of Medicine, Busan 49267, Korea; efmsungmo@hanmail.net; 2 Department of Family Medicine, Kosin University Gospel Hospital, Kosin University College of Medicine, Busan 49267, Korea

**Keywords:** fibromyalgia, cardiometabolic profiles, arterial stiffness, atherosclerosis

## Abstract

Background: The present study aimed to evaluate the association between FM and cardiometabolic risk factors and carotid arterial stiffness in FM patients. Methods: The cardiometabolic risk profile was defined based on the Adult Treatment Panel III panel. Carotid intimal media thickness (cIMT) and arterial stiffness were assessed using high-resolution ultrasonography. Multivariate logistic analysis was performed to estimate the association between FM and cardiometabolic risk factors. We used a general linear regression to compare the cIMT and carotid beta-index between the participants with and without FM. Pearson’s coefficient was calculated to evaluate the potential correlation between cardiometabolic risk profiles, cIMT, and arterial stiffening in FM. Results: FM participants showed a higher risk of central obesity (odds ratio [OR] = 3.21, 95% confidence interval [CI] 1.49, 6.91), high triglyceride (OR = 4.73, 95% CI 2.29, 9.79), and impaired fasting glucose (IFG) (OR = 4.27, 95% CI 2.07, 8.81) compared to the control group. The FM group exhibited higher beta-index values than the control group (*p* = 0.003). Although IFG and triglyceride glucose index showed a tendency to correlate with the beta-index, statistical significance was not observed. Conclusions: FM was associated with an increased risk of central obesity, high triglyceride levels, and IFG. Furthermore, advanced arterial stiffness of the carotid artery was observed in FM, which might be correlated with insulin resistance.

## 1. Introduction

Fibromyalgia (FM) is characterized by chronic widespread pain accompanied by depressive mood, sleep disturbances, fatigue, and cognitive impairment [1]. Although its pathophysiology has not been fully elucidated, the augmentation of pain and sensory processing plays a central role in the development of chronic pain and other concomitant symptoms of FM [2]. Relevant to chronic pain and accompanying symptoms, such as fatigue and sleep impairment, individuals with FM are in a state of physical inactivity and sleep deprivation that could predispose them to metabolic dysregulation.

Accumulating evidence indicates that FM is associated with cardiometabolic risk factors. In a study in the United States (US), patients with FM had a higher risk for central obesity and elevated glycosylated hemoglobin, serum triglyceride, and systolic (SBP) and diastolic blood pressure (DBP) than healthy controls [3]. Another study in Spain showed that FM patients were more likely to have central obesity and reduced cardiopulmonary function compared to those without FM [4]. In addition, a nationwide study in Taiwan suggested that individuals with FM were at a greater risk for cardiometabolic comorbidities, including hypertension, diabetes, and dyslipidemia, and cardiovascular diseases (CVDs) [5]. Decreased blood pressure variability, an indicator of cardiovascular morbidity, was also observed among FM participants in a small study that utilized ambulatory blood pressure measurement [6].

In addition to these conventional cardiometabolic risk profiles, arterial stiffness, which reflects the progression of atherosclerosis, has been recognized as a contributing factor to the increased risk of CVDs [7,8]. Measuring the pulse wave velocity or the aortic stiffness index, previous studies reported that individuals with FM were more likely to show advanced arterial stiffening compared with healthy participants [9,10,11,12,13]. Although the carotid-femoral pulse wave velocity is a standard technique to assess arterial stiffness [14], carotid arterial stiffness is also a useful predictor for the risk of CVDs [7,8]. However, despite the predictive value of carotid arterial stiffness in the development of CVDs [15], there is a knowledge gap in the association between FM and carotid arterial stiffness. Furthermore, because most studies assessing the risk of CVDs among FM patients only used either conventional risk profiles or biomechanical parameters of the arterial wall, the relationship between cardiometabolic factors and carotid arterial stiffness is still an area of uncertainty in patients with FM. 

In this regard, the present study evaluated the association between FM and cardiometabolic risk profiles and carotid arterial stiffness. Furthermore, the contribution of cardiometabolic risk factors to the biomechanical properties of the carotid artery was estimated using correlation analysis. 

## 2. Materials and Methods

### 2.1. Study Participants

This retrospective cross-sectional study included participants aged ≥19 years who visited a single university rheumatology outpatient clinic between January 2018 and December 2018. A total of 58 participants with FM with available information on cardiometabolic risk profiles were identified. The diagnosis of FM was made based on the revised American College of Rheumatology criteria [1]. In addition, 158 healthy controls who attended annual health check-ups at the Department of Disease Prevention and Health Promotion without evidence of inflammatory rheumatologic diseases were included in the study. The following exclusion criteria were applied to all participants: (i) autoimmune inflammatory conditions, (ii) malignancies, (iii) abnormalities in thyroid function, and (iv) acute coronary syndrome or stroke. Finally, 216 participants were included in the analysis. All study protocols complied with the Declaration of Helsinki, and written informed consent was waived. This study was reviewed and approved by the Institutional Review Board of Kosin University Medical School (KUGH-2020-05-023, 12 May 2020).

Based on the test statistics suggested by Woodward [16] and the prevalence of metabolic syndrome (MetS) in a previous study (prevalence of Mets was 0.21 in FMS and 0.04 in control) [3], we estimated that 58 participants would be necessary for each FMS and control group to ensure the sufficient power (≥80%) of a 2-sided significance test with an α = 0.05.

### 2.2. Data Collection and Measurements

Information on anthropometric measurements and health behaviors were collected using face-to-face interviews by trained medical assistants. We verified the information regarding anthropometric measurements (height and weight) and health behaviors (alcohol consumption and smoking status) using electronic medical records after the interview had been completed. Two independent physicians gathered data on hypertension, diabetes, and dyslipidemia by reviewing the electronic medical records. Hypertension was defined as SBP ≥ 140 mmHg, DBP ≥ 90 mmHg, or use of antihypertensive medication. Diabetes was defined as a fasting glucose level ≥ 100 mg/dL or use of anti-diabetic medications. Participants with a low-density lipoprotein (LDL) cholesterol level ≥ 130 mg/dL, high-density lipoprotein (HDL) cholesterol level < 40 mg/dL, triglyceride level ≥ 150 mg, or who were using lipid-lowering medications were defined as having dyslipidemia. 

Smoking status (smokers and non-smokers) and alcohol consumption (yes or no) were categorized into two groups. Body mass index (BMI) was calculated as kg/m^2^ and further categorized into three groups based on criteria tailored to the Asian population (<23.0, 23.0–24.9, and ≥25 kg/m^2^). Waist circumference (WC) was measured at the midpoint between the bottom of the rib cage and the top of the lateral border of the iliac crest and information on WC was only available among 187 participants.

Venous blood samples were collected after at least 8 h of fasting and analyzed at a diagnostic laboratory where regular quality control for laboratory tests are performed by an external quality assessment program. The serum triglyceride, HDL, and LDL cholesterol, and fasting glucose levels were measured using an enzymatic method, a two-reagent homogenous method, and the hexokinase G-6-PDH method, respectively, with an au 5800 Analyzer (Beckman Coulter, Brea, CA, USA). The immunoturbidimetry method was used to measure high-sensitivity C-reactive protein (hs-CRP) with a Cobas8000 (Roche, Mannheim, Germany). 

### 2.3. Ultrasound Examination for Carotid Plaque and Arterial Stiffness

Participants were examined in a supine position with their neck extended and their chin tilted 45° away from the examination side. A single operator scanned the right common carotid arteries of all participants using a high-resolution beta mode system (Aloka ProSound Alpha 7; Hitachi-Aloka Medical, Mitaka, Japan) equipped with a 13-MHz linear transducer in FM group. An ultrasound examination was performed by another examiner in the control group. To minimize inter-operator variability, the same ultrasound machines were used for the examination, and the calibration program to adjust inter-operator variability was embedded in the machines. The carotid intimal medial thickness (cIMT) was measured 2 cm distal to the bifurcation of the common carotid artery, and the thickness was determined by assessing the distance between the media-adventitia and the intima-lumen.

**Figure 1:**
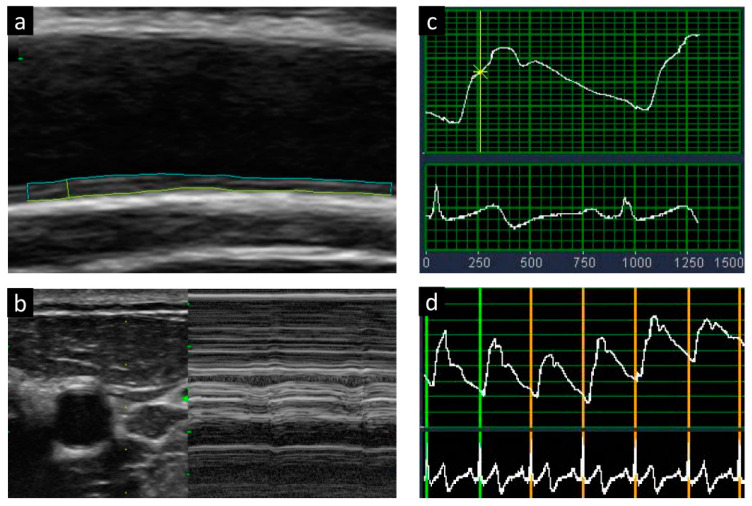
Measurements of carotid intimal media thickness (**a**,**b**) and arterial stiffness with the use of beta-index (**c**,**d**).

Carotid arterial stiffness was measured using a semiautomated echo-tracking technique, and simultaneous electrocardiography-gated time-related waveforms were obtained. Changes in the vessel wall diameter were automatically tracked in real-time, and five consecutive waveforms were recorded, taking the breathing effect into account. The following formula was used to calculate the carotid F: β = ln(SBP/DBP)/[(end-systolic diameter [ESD] − end-diastolic diameter [EDD])/EDD] [17]. The end-systolic diameters and EDDs were defined as the maximum and minimum common carotid artery diameters, respectively. The participants’ SBP and DBP were assessed using a standard mercury sphygmomanometer (Baum Co., Inc., Copiague, NY, USA) after at least 15 min of rest (Figure 1).

### 2.4. Definition of Cardiometabolic Risk Profiles and Insulin Resistance

Obesity and central obesity were defined as BMI ≥ 25 m/k^2^ and WC ≥ 80 cm, respectively, according to the specific criteria for the Asian population [18]. Based on the Adult Treatment Panel III [19], high blood pressure was defined as SBP ≥ 130 mmHg, DBP ≥ 85 mmHg, or antihypertensive medication use. High triglyceride and high LDL cholesterol levels were defined as serum triglyceride ≥ 150 mg/dL and serum LDL cholesterol ≥ 130 mg/dL, respectively. The threshold for low HDL cholesterol level was <40 mg/dL. Regardless of lipid levels, participants on lipid-lowering agents were classified as high triglycerides, high LDL cholesterol, and low HDL cholesterol levels. Impaired fasting glucose (IFG) was defined as fasting glucose ≥ 100 mg/dL or being on anti-diabetic medications. Participants who showed hs-CRP ≥ 0.75 mg/dL were defined as being in a state of inflammation. We generated a composite cardiometabolic risk score composed of BMI and blood pressure, serum triglyceride, LDL cholesterol, HDL cholesterol, IFG, and hs-CRP levels.

Insulin resistance was measured indirectly using the triglycerides/glucose index (TGI), which is strongly correlated with homeostatic model assessment for insulin resistance (HOMA-IR) [20].

### 2.5. Statistical Analysis

The study participants’ general characteristics were compared between FM and healthy controls using the *t*-test for continuous variables (age) and the chi-square test for categorical variables (smoking status, alcohol consumption, hypertension, diabetes, and BMI). An age- and multivariable-adjusted (age, smoking status, and alcohol consumption) general linear regression analysis was performed to estimate and compare the cardiometabolic profiles (BMI, WC, SBP, DBP, serum triglyceride, LDL cholesterol, HDL cholesterol, fasting glucose, and hs-CRP) between the FM and control groups. Data are presented as the mean and 95% confidence interval (CI).

We performed a multivariate logistic analysis to evaluate the association between FM and cardiometabolic risk factors (BMI ≥ 25 m/kg^2^, WC ≥ 80 cm, SBP ≥ 130 mmHg, DBP ≥ 85 mmHg, or antihypertensive medication use; triglyceride ≥ 150 mg/dL, LDL cholesterol ≥ 130 mg/dL, HDL cholesterol level < 40 mg/dL, fasting glucose ≥ 100 mg/dL or the use of anti-diabetic medications, and hs-CRP ≥ 0.75 mg/dL) and a composite score for cardiometabolic risk. In addition, the cIMT and carotid beta-index between participants with and without FM was compared using general linear regression analysis. Pearson’s coefficient was calculated to evaluate the potential correlation between cardiometabolic risk profiles, cIMT, and arterial stiffening in FM participants.

Two-tailed tests were performed in all analyses, and *p*-values of <0.05 were considered statistically significant. All analyses were performed using IBM SPSS Statistics for Windows v24.0 (IBM Corp., Armonk, NY, USA).

## 3. Results

### 3.1. General Characteristics of the Study Participants

The general characteristics of the study participants are listed in Table 1. The FM group had a lower mean age compared to the control group (53.2 ± 12.2 vs. 57.4 ± 9.7 years). FM patients were more likely to consume alcohol; the prevalence of dyslipidemia was higher than that in the control group. The other descriptive variables were not significantly different between the two groups.

### 3.2. Comparison of the Cardiometabolic Profiles and a Composite Score of Cardiometabolic Risk

The comparison of the cardiometabolic profiles between participants with FM and without FM is shown in Table 2. Although FM patients had comparable BMI to non-FM participants, those in the FM group had a higher WC (86.6 cm [95% CI 84.2, 89.0 cm] vs. 81.2 cm [95% CI 79.7, 82.8 cm], *p* < 0.001). In the age-adjusted analysis, participants with FM had higher serum triglyceride levels (174.0 mg/dL [153.2, 194.8 mg/dL] vs. 106.9 mg/dL [94.8, 119.1 mg/dL], *p* < 0.001) and blood pressure levels than those without FM. The HDL cholesterol level was lower in the FM group than in the control group in the age-adjusted analysis; however, the difference was not significant in the multivariable analysis. No significant differences in the blood pressure, LDL cholesterol, and hs-CRP levels were observed between the two groups.

A composite score of cardiometabolic risk was significantly higher in participants with FM than in those without FM after the adjustment for potential covariates (3.22 [95% CI 2.92, 3.62] in FM, 2.64 [95% CI 2.43, 2.85] in non-FM).

### 3.3. The Risk for Cardiometabolic Risk Factors

**Figure 2:**
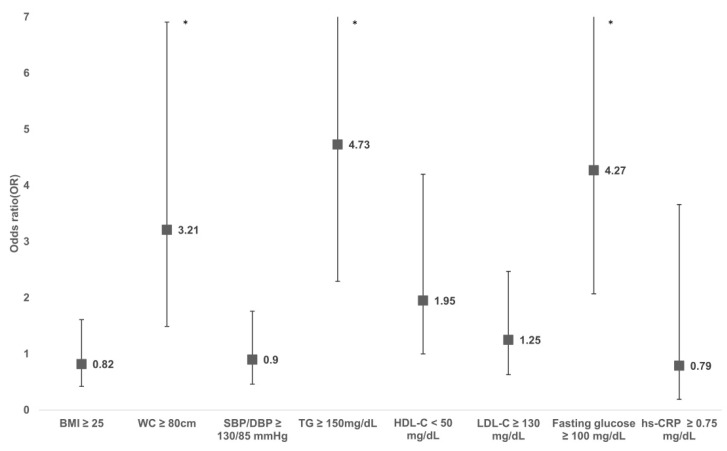
Adjusted odds ratio of cardiometabolic risk factors for adults with FMS compared with those without FMS. Odds ratio was calculated using multi-variable logistic regression adjusting for age, smoking, and alcohol consumption. Asterisk (*) represents *p* ≤ 0.05.

The risk for cardiometabolic risk factors is shown in Figure 2. Participants with FM had a significantly higher risk of central obesity (odds ratio [OR] = 3.21 [95% CI 1.49, 6.91]), high triglyceride (OR = 4.73 [95% CI 2.29, 9.79]), and IFG (OR = 4.27 [95% CI 2.07, 8.81]) compared to those without FM. While there was a lower HDL cholesterol level in FMS patients, this was not statistically significant (OR = 1.95 [95% CI 1.00, 4.20]). There was no substantial difference in the risk of obesity, high blood pressure, and high LDL cholesterol between the FM and non-FM groups.

### 3.4. Carotid Intimal Media Thickness and Beta-Index

Although FM patients showed higher cIMT than the control group in the age-adjusted model, this difference was no longer significant after adjusting for other covariates, including smoking and alcohol consumption. When we compared the carotid beta-index, the FM group exhibited higher beta-index values than the control group across the analysis models (Table 3).

### 3.5. Correlation between Cardioembolic Risk Profiles and cIMT and Beta Index

Pearson’s coefficient was calculated to explore the link between cardioembolic risk profiles and cIMT and beta index among the participants with FM (Table 4). Although IFG (*p* = 0.057) and TGI (*p* = 0.072) tended to be associated with the beta-index, statistical significance was not observed. No cardiometabolic risk factors were associated with cIMT.

## 4. Discussion

The present study showed that individuals with FM had a higher risk of cardiometabolic profiles and advanced arterial stiffness compared to the control group. For specific metabolic risk factors, the risk of central obesity, a high triglyceride level, and IFG were significantly higher in FM patients than in those without. Although FM showed a tendency to increase the risk of low HDL cholesterol, this was not statistically significant. Despite the advanced arterial stiffness, the cIMT in the individuals with FM was not significantly different in those without FM. While IFG and TGI showed a positive correlation with the carotid beta-index, this was not statistically significant.

Despite having similar BMIs, the participants with FM had a higher risk of central obesity compared with the healthy controls, and this finding was consistent with a previous study in the US [3]. In that study, a higher WC and waist–hip ratio, but not BMI, were observed in the patients with FM compared to the control group. A Spanish study also demonstrated that central obesity was higher among participants with FM than in the control group [4]. Central obesity is a marker of excess visceral adiposity, which plays an important role in the development of CVD and diabetes through insulin resistance and inflammation [21,22]. Furthermore, an earlier US study demonstrating that normal-weight central obesity was a better predictor of CVD mortality than BMI-defined obesity also supports elevated WC as an independent risk factor for CVD [23]. The positive association between FM and central obesity was not attributable to age, smoking, or alcohol consumption, suggesting that FM with central obesity might be at greater risk for CVD compared to the general population.

The present study showed that the risk for high TG was 4.7 times higher in the FM patients than in the healthy controls. Accumulating evidence from previous studies has indicated that FM is associated with increased TG levels and dyslipidemia rate [3,4,5]. Together with central obesity, high TG is a key marker of insulin resistance, which contributes to the development of atherosclerosis and CVD [24,25]. In addition, higher TGI observed in participants with FM compared to non-FM participants provided biochemical support to the idea that FM was associated with increased insulin resistance. A recent study showing the association between FM and insulin resistance supports our findings [26]. However, a few studies have reported no significant association between FM and high TG [6,27]. The possible reason for these conflicting results could be the different assessment methods for insulin resistance among studies. Although in our study, TGI was considered a marker for insulin resistance [20], there might be some heterogeneities in reflecting insulin resistance when measured in terms of TGI compared with HOMA-IR, which considers the serum insulin level. Therefore, further studies on insulin resistance using HOAM-IR are warranted to test the association between FM and insulin resistance. Moreover, differences in study design and sample size might be related to the discrepancies, and further longitudinal studies are warranted to confirm the findings of the current study.

Participants with FM had a higher risk of IFG compared to healthy controls. In line with our study, higher A1c levels and prevalence of diabetes were observed in FM patients compared to those in the control group [3,5,26], while another study failed to show a significant association between FM and IFG [6]. Contrary to previous studies, our study showed that IFG was independently associated with FM after accounting for potential confounders, such as smoking and alcohol consumption, in the analysis. In addition, an elevated surrogate marker level of insulin resistance (TGI) in FM participants, at least partly, supported the negative influence of FM on serum glucose levels.

Although FM was not associated with cIMT plaque, a higher carotid beta-index was observed in FM patients than in the control group, suggesting that the elastic property of the arterial wall in patients with FM was changed without causing any visible structural changes on ultrasound [28]. In line with our study, significantly elevated arterial stiffness was found in individuals diagnosed with FM with different measurement modalities and arterial location [9,10,11,12,13]. Advanced carotid arterial stiffness, which reflects a decreased elastin content and an increased content of collagen [29], can be used as an indicator for atherosclerotic change [30] and carotid remodeling [31], and this biomechanical alteration is associated with the risk of CVD and CVD mortality [7,8]. No significant association between FM and cIMT could be concluded as cIMT is not a reliable marker to reflect atherosclerosis in FM and more time was needed to observe a significant change in visual structural change in the carotid arterial wall among FM patients. Nevertheless, considering the easy accessibility and low inter-operator variability of carotid echo-tracking, assessing carotid arterial stiffness might be a useful tissue biomarker to identify patients with FM who are at a high risk for CVD.

In the analysis of the link between cardiometabolic risk profiles and arterial stiffness, IFG and TGI could be correlated with advancement in arterial stiffness in FM, although statistical significance was not reached. Previous studies on the association between IFG and atherosclerosis have reported inconsistent results. While IFG has been suggested as a marker for subclinical atherosclerosis [32,33] and unrecognized myocardial infarction [34], other studies have argued that IFG without impaired glucose tolerance was not associated with atherosclerosis [35,36]. Compared to most previous studies that primarily focused on the general population, the association of IFG with parameters of atherosclerosis might be different in participants with FM. Unlike IFG, considerable evidence has consistently supported the association between insulin resistance and atherosclerosis [37]. In addition, our study might partly contribute to its possible association with FM patients as well. However, because the sample size of the present study was relatively small, further larger-scale studies are necessary to strengthen the observed correlation and elucidate other related mechanisms of advancement in carotid arterial stiffness.

Several plausible mechanisms could be hypothesized to explain the observed associations. First, the enhanced sympathetic activity caused by FM could induce both insulin resistance, which has a detrimental effect on cardiometabolic profiles [38], and endothelial dysfunction, which is associated with the progress of atherosclerosis [39,40]. The findings of elevated norepinephrine and epinephrine ratios in FM and metabolic syndrome in a previous study support this hypothesis [3]. In addition, sympathetic activation leads to elevated blood pressure that is attributable to the progression of atherosclerosis in the carotid artery [41]. Despite these potential mechanisms, the present study failed to demonstrate a significant association between FM and the risk of high blood pressure, suggesting the involvement of other mechanisms in subclinical atherosclerosis in FM participants. A recent experimental study suggested that high blood pressure derived from enhanced sympathetic tone modulated the hematopoietic system, thereby contributing to the development of atherosclerosis as an alternative pathway linking FM and atherosclerosis [42]. Second, the dysregulation of the hypothalamus–pituitary–adrenal axis related to chronic pain and/or stress could be attributable to insulin resistance [43] and FM [44]. The shared involvement of hypothalamus–pituitary–adrenal axis impairment in FM and insulin resistance might play a role in the association of FM with cardiometabolic profiles and arterial stiffness. Third, chronic pain in FM could be implicated in the endothelial dysfunction of the carotid artery. FM patients with higher visual analog score (VAS) pain scores were more likely to have impaired endothelial function compared to those with lower VAS [45]. Fourth, the sedentary lifestyle of FM patients could be associated with detrimental changes in cardiometabolic profiles and subclinical atherosclerosis. Physical inactivity and sedentary hours are reported to be associated with an increased risk of obesity in FM patients [46], and elevated BMI that is associated with physical inactivity might play a significant role in the negative consequences of cardiometabolic health and atherosclerosis.

The clinical implication of our findings is that physicians who treat FM patients should be aware of the elevated risk of central obesity, high triglyceride levels, IFG, and arterial stiffness in FM. Thus, if physicians encounter FM patients at high risk for CVD, such as those with a family history of CVD or who complain of chest pain or dyspnea on exertion, immediate screening and management for CVD might be necessary.

The current study had several limitations. First, because the cross-sectional study design could not account for temporality, caution should be exercised when interpreting the causality of the observed associations. Second, all study participants were Korean women; thus, the generalizability of our study findings to men or other ethnicities could be limited. Third, although we controlled for potential confounders in the analysis models, residual confounders, such as duration of diabetes, physical activities, and socioeconomic status, might have affected the observed findings. In addition, smoking status and alcohol consumption were categorized dichotomously, and residual confounding effects related to these variables might exist. Fourth, because of the limited information on disease severity among patients with FM, it was not feasible to evaluate the association of FM-related disease activities, such as impairment in daily activities and VAS of pain, with cardiometabolic profiles and arterial stiffness. Future studies should consider these factors to shed light on the impact of FM severity on the cardiometabolic health of patients with FM. Fifth, despite the use of the same ultrasound machines and calibration program to minimize inter-operator variability, inter-observer bias might have affected the observed findings. Despite these limitations, the present study was noteworthy because it provided evidence of elevated cardiometabolic risk factors among patients with FM.

## 5. Conclusions

The present study revealed that FM was associated with an increased risk of central obesity, high triglyceride levels, and IFG. Furthermore, advanced arterial stiffness of the carotid artery was observed in participants with FM, and this advancement might be correlated with insulin resistance. Additional longitudinal studies are warranted to replicate and confirm the observed findings, and strategies for risk stratification according to cardiometabolic profiles might be necessary for FM patients.

## Figures and Tables

**Table 1:** General characteristics of study participants.

	FMS (*n* = 58)	Control (*n* = 158)	*p*-Value
Age, year	53.2 (12.2)	57.4 (9.7)	0.011
Smoking status			<0.001
Non-smoker	45 (77.6)	150 (94.9)	
Smoker	13 (22.4)	8 (5.1)	
Alcohol consumption			0.008
Yes	47 (81.0)	98 (62.0)	
No	11 (19.0)	60 (38.0)	
Hypertension			0.978
Yes	41 (70.7)	112 (70.9)	
No	17 (29.3)	46 (29.1)	
Diabetes			0.537
Yes	50 (86.2)	141 (89.2)	
No	8 (13.8)	17 (10.8)	
Dyslipidemia			0.028
Yes	34 (58.6)	66 (41.8)	
No	24 (41.4)	92 (58.2)	
Body mass index, kg/m^2^	24.4 (3.6)	24.0 (3.3)	0.456 0.455
<23.0	22 (39.9)	61 (38.6)	
23.0–24.9	12 (20.7)	44 (27.8)	
≥25.0	24 (41.4)	53 (33.5)	

Data are presented as mean [standard deviation (SD)] or number (%). *p*-value was obtained using *t*-test for continuous variable and chi-square test for categorical variables respectively.

**Table 2:** Cardiometabolic profiles among female fibromyalgia syndrome (FMS) compared with those without FMS.

	Age-Adjusted Analysis		*p*-Value	Multivariable-Adjusted Analysis		*p*-Value
	FMS (*n* = 58)	Control (*n* = 158)		FMS (*n* = 58)	Control (*n* = 158)	
Measures of obesity						
Body mass index, kg/m^2^	24.3 (23.4, 25.2)	24.0 (23.5, 24.6)	0.610	24.1 (23.2, 25.1)	24.1 (23.6, 24.6)	0.934
Waist circumference, cm	87.0 (84.7, 89.3)	81.0 (79.5, 82.6)	<0.001	86.6 (84.2, 89.0)	81.2 (79.7, 82.8)	<0.001
Blood pressure						
Systolic blood pressure, mmHg	124.0 (120.4, 127.6)	124.7 (122.5, 126.9)	0.748	124.1 (120.3, 127.9)	124.6 (122.4, 126.9)	0.819
Diastolic blood pressure, mmHg	74.7 (72.2, 77.2)	75.5(75.0, 77.0)	0.577	75.1(72.5,77.7)	75.4 (73.9, 76.9)	0.840
Lipid profiles						
Serum triglyceride, mg/dL	176.4 (156.7, 196.1)	106.1 (94.2, 117.9)	<0.001	174.0 (153, 2.194.8)	106.9 (94.8, 119.1)	<0.001
HDL cholesterol, mg/dL	52.8 (49.0, 56.7)	58.0 (55.7,60.3)	0.025	54.2 (50.2, 58.2)	57.5 (55.2, 59.9)	0.173
LDL cholesterol, mg/dL	114.0 (105.6, 122.4)	121.2 (116.2, 126.3)	0.150	115.4 (106.6, 124.3)	120.7 (115.5, 125.9)	0.329
Glucose						
Fasting glucose, mg/dL	105.5 (100.7, 110.3)	91.1 (88.2, 94.0)	<0.001	107.1 (102.1, 112.2)	90.5 (87.5, 93.4)	<0.001
Inflammation						
hs-CRP	0.17 (0.06, 0.27)	0.16 (0.09, 0.23)	0.944	0.16 (0.05, 0.27)	0.16 (0.10, 0.23)	0.977
A composite score of cardiometabolic risk	3.31 (2.98, 3.64)	2.63 (2.43, 2.83)	0.001	3.22 (2.92, 3.62)	2.64 (2.43, 2.85)	0.003

Data was presented with mean and 95% confidence interval. *p*-value was calculated with general linear regression analysis adjusting for age, smoking status, and alcohol consumption. Waist circumference was measured among 187 participants (57 of FMS, 130 of control).

**Table 3:** Cardiometabolic profiles among female fibromyalgia syndrome (FMS) compared with those without FMS.

	Age-Adjusted		*p*-Value	Multi-Variable Adjusted		*p*-Value
	FMS (*n* = 58)	Control (*n* = 158)		FMS (*n* = 58)	Control (*n* = 158)	
cIMT	0.70 (0.65, 0.75)	0.65 (0.61, 0.68)	0.068	0.70 (0.64, 0.75)	0.65 (0.62, 0.68)	0.145
Carotid beta-index	10.6 (9.6, 11.5)	8.1 (6.8, 9.5)	0.004	10.6 (9.7, 11.5)	8.0 (6.6, 9.4)	0.003

cIMT: Carotid intimal media thickness. Data were presented with mean and 95% confidence interval. *p*-value was calculated with general linear regression analysis adjusting for age, smoking status, and alcohol consumption. Carotid beta-index was measured among 83 participants (56 of FMS, 27 of control).

**Table 4:** Correlation between cardio-metabolic profiles and carotid intimal medial thickness and carotid beta-index in participants with FM.

	Carotid Intimal Medial Thickness		Carotid Beta-Index	
	r	*p*-Value	r	*p*-Value
Body mass index, kg/m^2^	−0.006	0.964	0.128	0.365
Waist circumference, cm	0.004	0.976	0.163	0.247
Serum triglyceride, mg/dL	−0.17	0.228	0.149	0.29
HDL cholesterol, mg/dL	0.064	0.652	0.037	0.795
LDL cholesterol, mg/dL	−0.067	0.639	0.134	0.342
Fasting glucose, mg/dL	0.05	0.726	0.266	0.057
Triglyceride glucose index	−0.093	0.51	0.251	0.072
hs-CRP	−0.032	0.822	0.17	0.227

*p*-value was calculated with Pearson correlation analysis adjusting for age, smoking status, and alcohol consumption.

## Data Availability

The data are available from the authors upon reasonable request and with permission of the Institutional Review Board of Kosin University Medical School.
